# Chemical Diversity and Biological Properties of Secondary Metabolites from Sea Hares of *Aplysia* Genus

**DOI:** 10.3390/md14020039

**Published:** 2016-02-19

**Authors:** Renato B. Pereira, Paula B. Andrade, Patrícia Valentão

**Affiliations:** REQUIMTE/LAQV, Laboratory of Pharmacognosy, Department of Chemistry, Faculty of Pharmacy, University of Porto, R. Jorge Viterbo Ferreira No. 228, 4050-313 Porto, Portugal; ren.pereira@gmail.com (R.B.P.); pandrade@ff.up.pt (P.B.A.)

**Keywords:** mollusks, sea hares, *Aplysia*, secondary metabolites, biological properties

## Abstract

The marine environment is an important source of structurally-diverse and biologically-active secondary metabolites. During the last two decades, thousands of compounds were discovered in marine organisms, several of them having inspired the development of new classes of therapeutic agents. Marine mollusks constitute a successful phyla in the discovery of new marine natural products (MNPs). Over a 50-year period from 1963, 116 genera of mollusks contributed innumerous compounds, *Aplysia* being the most studied genus by MNP chemists. This genus includes 36 valid species and should be distinguished from all mollusks as it yielded numerous new natural products. *Aplysia* sea hares are herbivorous mollusks, which have been proven to be a rich source of secondary metabolites, mostly of dietary origin. The majority of secondary metabolites isolated from sea hares of the genus *Aplysia* are halogenated terpenes; however, these animals are also a source of compounds from other chemical classes, such as macrolides, sterols and alkaloids, often exhibiting cytotoxic, antibacterial, antifungal, antiviral and/or antifeedant activities. This review focuses on the diverse structural classes of secondary metabolites found in *Aplysia* spp., including several compounds with pronounced biological properties.

## 1. Introduction

Marine natural products (MNPs) have attracted increasing research attention during the last two decades [1,2]. To date, over 25,000 new compounds from marine organisms have been reported and characterized, most of them exhibiting a wide range of biological activities, playing an important role in the discovery of leads for the development of drugs for the treatment of human diseases [3]. It is estimated that approximately 56% of the new active marine natural products present anti-cancer activity, followed by 13% with antibacterial properties [4]. As reported by Hu [5], approximately 75% of the compounds were isolated from marine invertebrates, such as mollusks, sponges and echinoderms. Among the Mollusca phylum, 116 genera in particular contributed to the discovery of new compounds over a 50-year period from 1963 [2,6]. Indeed, 18 marine mollusk–derived compounds presenting anti-cancer properties are in phase I, II or III of drug development [7]. One example is dolastatin 10, an antineoplastic peptide first obtained from the Indian Ocean sea hare *Dolabella auricularia* Lightfoot [8] and later found to be a product of the gastropod’s cyanobacterial (*Symploca* sp.) diet [9,10]. In 2011, brentuximab vedotin (Adcetris^®^), an antibody-dolastatin 10 conjugate, was approved by the FDA for the treatment of Hodgkin’s lymphoma [11,12].

Sea hares are considered as shell-less mollusks, as they have only a degenerated shell in their mantle cavity, exposing their naked and soft bodies to the surroundings [13]. The absence of a shell as physical protection is compensated by several behavioral, anatomical and physiological adaptations [13,14]. However, the effective defense mechanism displayed by these organisms is the chemical and behavioral one, releasing a purple ink and opaline when attacked by predators [13,15,16]. These secretions contain some bioactive molecules acting by different mechanisms, such as feed stimulants (e.g., taurine), feed deterrents, and aversive compounds [6,13]. Among all mollusks, the sea hares of the *Aplysia* genus are the most studied by the MNP chemists [2,6]. According to the World Register of Marine Species (WoRMS) database [17], this genus includes 36 valid species and yielded, until 2011, 58 new natural products, *Aplysia dactylomela* Rang being the source of almost half of them [6,18]. *Aplysia* sea hares are herbivorous (feeding predominantly on red algae) with the ability to store sequestered bioactive algal metabolites in a specialized digestive gland [6,19]. However, the compounds isolated from *Aplysia* sea hares are either algal natural products obtained from the diet and slightly modified derivatives of them, or they are made *de novo* by direct synthesis [20].

The first approach on secondary metabolites of a shell-less gastropod dates back to 1963, when Yamamura and Hirata isolated different sesquiterpenes from specimens of *Aplysia kurodai* Baba [21]. In 2006, Kamiya and co-authors briefly reviewed some bioactive molecules from sea hares [22]. Since then, a huge number of molecules have been discovered, especially from sea hares of the *Aplysia* genus. More recently, two articles appeared establishing the fatty acid [23] and carotenoid [24] composition of different *Aplysia* sea hares by GC-MS and LC-DAD, respectively. However, the current review emphasizes the secondary metabolites effectively isolated from sea hares of the *Aplysia* genus and their biological properties. For this purpose, the compounds were systematized according to their chemical class in macrolides, C_15_-acetogenins, sterols, monoterpenes, sesquiterpenes, diterpenes and triterpenes, among other metabolites.

## 2. Macrolides

**Figure 1:**
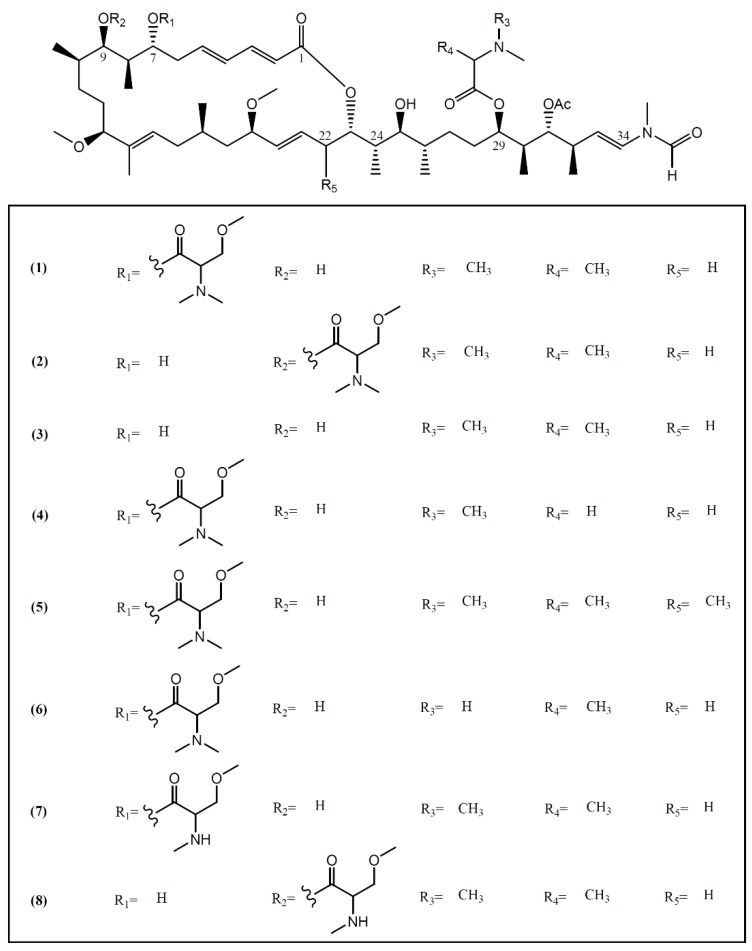
Structures of aplyronines isolated from *A. kurodai*.

Most macrolides are known as a group of broad-spectrum antibiotics used in the treatment of common bacterial and fungal infections. Some of them, especially from marine sources, have been reported as interesting anti-cancer agents [25]. Marine macrolides are characterized by a highly oxygenated polyene backbone and a macrocyclic lactone [26]. The discovery of these typical structures in sea hares of the *Aplysia* genus started in 1993, when Yamada and collaborators [27] isolated aplyronines A–C (**1**–**3**) (Figure 1) from the fractionation of a lipophilic extract of *A. kurodai* collected on the Pacific coast of Mie Prefecture (Japan). Aplyronine A (**1**), as well as its congeners aplyronines B (**2**) and C (**3**)**,** proved to be *in vitro* potent antitumor substances, displaying IC_50_ values of 0.45, 2.9 and 22 nM against a human cervical carcinoma cell line (HeLa S_3_), respectively [28]. Aplyronine A (**1**) proved to be much more cytotoxic than aplyronine C (**3**), a derivative of **1** lacking the C-7 trimethylserine ester moiety [29]. *In vivo* studies developed by the same group showed that aplyronine A (**1**) exhibited exceedingly potent antitumor activity in mouse xenograft models (e.g., P388 murine leukemia, Lewis lung carcinoma, Ehrlich carcinoma, CT26 colon carcinoma and B16 melanoma) [27]. Saito *et al.* [30], aiming to establish the anti-cancer mechanism of aplyronine A (**1**), studied its ability to interact with actin, the most abundant protein in the eukaryotic cytoskeleton, responsible for the regulation of various cell functions such as muscle contraction, cell mobility and cell division [31]. Results indicated that aplyronine A (**1**) has an actin-depolymerizing effect, inhibiting the velocity and the degree of actin polymerization by forming a 1:1 complex with monomeric actin. They also suggested that the C24-C34 side-chain (Figure 1) but not the macrolide ring binds to actin to depolymerize the filament [30]. More recent work developed by Kita and collaborators [32] demonstrated that, in addition to the action of the side-chain, the actin-aplyronine A complex synergistically binds to tubulin at very low concentrations, inhibiting tubulin polymerization, and prevents spindle formation and mitosis in tumor cells. The same study showed that aplyronine C (**3**)**,** which inhibits actin polymerization *in vitro* to the same extent as aplyronine A (**1**), does not bind to tubulin, explaining the substantial differences in cytotoxicity effects of compound **1** and **3** in HeLa S_3_ cells reported above [27,32,33]. In addition, treatment with aplyronine A (**1**) at 1 nM led to potent caspase 3 activation in HeLa S_3_ cells, while treatment with aplyronine C (**3**) did not, even at 100 nM [33].

Aplyronines D–H (**4**–**8**), new congeners of aplyronine A (**1**), were later isolated by Ojika *et al.* [28] from specimens of *A. kurodai* collected in the same geographical area. The same team also evaluated the cytotoxicity of compounds **4**–**8** against HeLa S_3_ cells, having obtained IC_50_ values of 0.075, 0.18, 0.19, 0.12 and 9.8 nM, respectively [28]. Most of the new congeners showed comparable or higher activity than aplyronine A (**1**), indicating that the methylation on the carbon skeleton (C-22) or demethylation in the amino acid moieties (C-7 trimethylserine and C-29 dimethylalanine) enhances or does not affect the cytotoxicity [28].

As such, the C-7 trimethylserine ester moiety of aplyronine A (**1**) seems to be essential to the interaction with tubulin and in promoting the activation of caspase 3; however, studies involving aplyronines D (**4**), E (**5**) and F (**6**) which also contain the C-7 trimethylserine ester moiety would be interesting to confirm this interaction. As compounds **4**–**7** presented stronger cytotoxicity than aplyronine A (**1**), further investigations are required to establish a structural active relationship between the observed cytotoxicity and the binding of these aplyronines to actin/tubulin and the activation of caspase 3.

**Figure 2:**
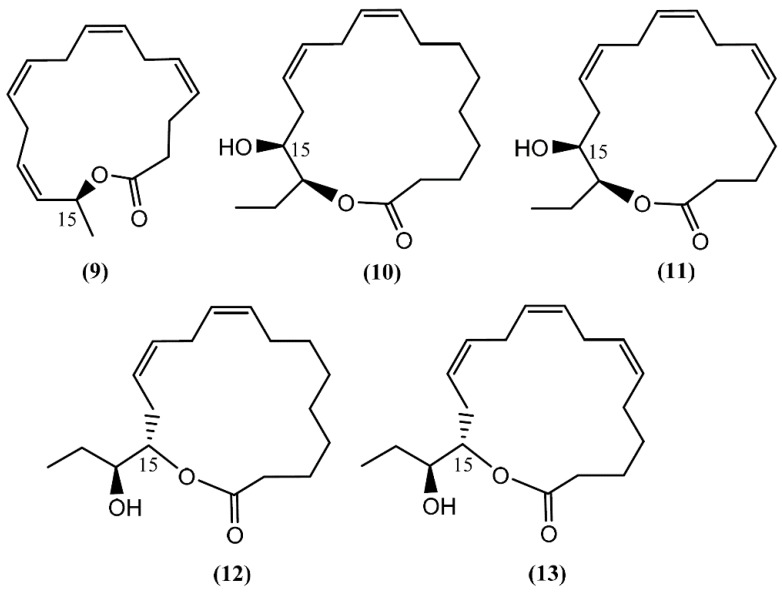
Structures of aplyolides isolated from *Aplysia depilans*.

Aplyolides are unusual molecules belonging to a small group of hydroxyl fatty acid lactones isolated from marine organisms [34]. Aplyolides A–E (**9**–**13**) (Figure 2), five unprecedented C_16_ and C_18_ fatty acid lactones, were isolated in 1997 from the external body parts of the sea hare *Aplysia depilans* Gmelin collected on the Atlantic coast, Asturias (Spain), and the Mediterranean coast, Naples (Italy). The absolute stereochemistry at C-15 was determined by Mosher's method after the opening of the lactone ring [35]. According to the study developed by Spinella *et al*. [35], the anatomical localization of aplyolides points to their potential biological role as defensive allomones and perhaps to the possibility of the sea hare to biosynthesize these lactones. It is interesting to note that compounds **9**–**13** were demonstrated to be ichthyotoxic to the mosquito fish *Gambusia affinis* at 10 ppm, whereas the corresponding methyl esters were completely inactive [35]. Although this group of molecules is associated with interesting biological activities, their paucity precludes more advanced studies.

## 3. C_15_-Acetogenins

**Figure 3:**
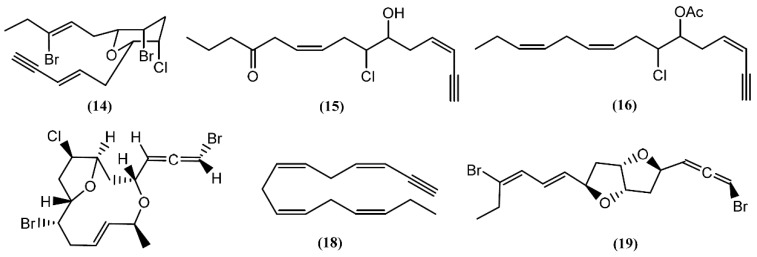
C_15_-Acetogenins isolated from sea hares of *Aplysia* genus.

Many acetogenins, namely C_15_-halogenated cyclic ethers, have been found in different *Aplysia* species. Most of these compounds have their origin in algae, especially those from the *Laurencia* and *Plocamium* genus, which these mollusks feed [20,36]. A number of closely related halogenated cyclic ethers are good inhibitors of drug metabolism, some presenting ichthyotoxic and antifeedant properties [37,38]. The first to be isolated was dactylyne (**14**) (Figure 3), in 1975, an acetylenic dibromochloro ether from the sea hare *A. dactylomela* [39]. Three years later, Kaul and Kulkarni [40] studied the pharmacological effect of this drug, demonstrating that it had no direct effect on the cardiovascular, respiratory, and central nervous systems of mice, rats, and guinea pigs. However, in rats, a 25 mg/kg intraperitoneal dose of dactylyne (**14**) inhibited pentobarbital metabolism, prolonging sleep time in mice by more than 10 h [40,41]. Its total synthesis was achieved by Gao and Murai in 1992 [42]. Chromatographic separations of the digestive and hermaphroditic glands’ organic extract of *Aplysia fasciata* Poiret led to the isolation of (3*Z*,9*Z)*-7-chloro-6-hydroxy-12-oxo-pentadeca-3,9-dien-1-yne (**15**) and (3*Z*,9*Z*,12*Z*)-6-acetoxy-7-chloro-pentadeca-3,9,12-trien-1-yne (**16**), two linear acetogenins [43] with no bioactivity described until now. Dactylallene (**17**) was found in the digestive gland of the *A. dactylomela* mollusk collected in the Canary Islands, Spain. It was revealed to be ichthyotoxic and displayed interesting antifeedant activity, suggesting a defensive role against predators [38]. A C_15_-acetogenin called laurencenyne (**18**) was isolated from the ethanol extract of *Aplysia punctata* Cuvier collected from the northwest coast of Sardinia (Italy) [44]. It was isolated earlier from *Laurencia okamurae* Yamada and, due to its structure, Kigoshi and collaborators [45] suggested laurencenyne (**18**) as a possible precursor of various nonterpenoid C_15_-compounds in the marine red algae of the genus *Laurencia*. Aplysiallene (**19**), a new bromoallene targeting Na^+^, K^+^-ATPase, was reported in *A. kurodai* and collected at the coast of Fukui (Japan) [46]. After being revised, the structure of aplysiallene (**19**) was found to match that of a bromoallene reported from *L. okamurae* [47,48].

**Figure 4:**
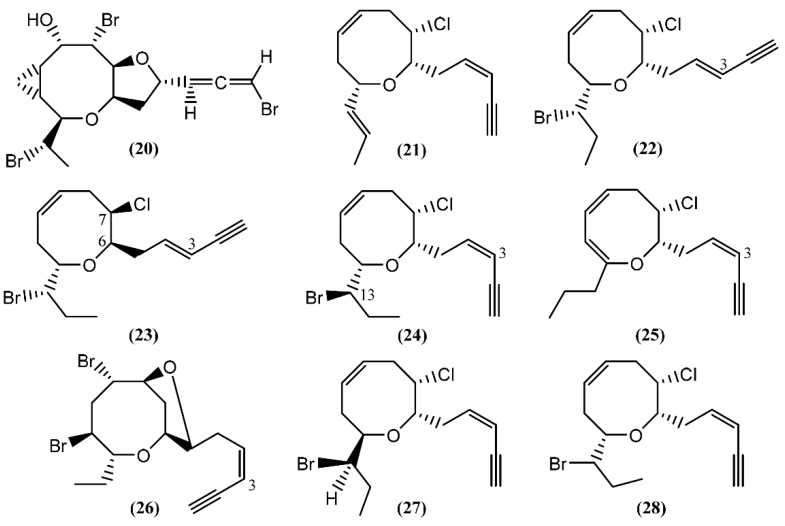
Eight-membered cyclic ether acetogenins isolated from sea hares of *Aplysia* genus.

Based on spectroscopic evidence and single-crystal X-ray analysis, Miyamoto *et al*. [49] elucidated the structure of aplyparvunin (**20**) (Figure 4), an ichthyotoxic acetogenin isolated from the Japanese marine mollusk *Aplysia parvula* Mørch. Other eight-membered cyclic ethers, namely (+)-laurenyne (**21**), (+)-3-*E*-pinnatifidenyne (**22**) and (−)-3*E*,6*R*,7*R*-pinnatifidenyne (**23**), were isolated from a South China Sea collection of the anaspidean mollusk *A. dactylomela* [50]. The (+)-Laurenyne (**21**) was previously isolated from another sea hare, *A. punctata* [44], and firstly isolated from the alga *Laurencia obtusa* (Hudson) Lamouroux [51]. This compound displayed toxicity toward brine shrimp, with an LC_50_ value of 467.0 µM [52]. The (3*Z*)-13-*epi*-Pinnatifidenyne (**24**), an isomer of **22** and **23**, was described in the digestive glands of *A. fasciata* [43]. The 3*Z*-Venustinene (**25**), a compound containing a novel propyl side-chain without bromine, was firstly isolated from *Laurencia venusta* Yamada [53] and posteriorly was found by Ioannou and colleagues in studies performed with *A. fasciata*, collected from the Alfacs Bay, Delta de l’Ebre (Spain) [43]. In 2005, McPhail and Davies-Coleman [54] described the isolation of (3*Z*)-bromofucin (**26**) from 49 specimens of the cosmopolitan sea hare *A. parvula*, collected in the Tsistsikamma National Park on the southeast coast of South Africa. It is of note that the majority of the ^1^H and ^13^C NMR data of (3*Z*)-bromofucin (**26**) were consistent with those of (3*E*)-bromofucin isolated from *Laurencia implicata* J.Agardh [55]. The occurrence of similar compounds in *Laurencia* algae indicated that these species are also the source of (3*Z*)-bromofucin (**26**) sequestered by *A. parvula* from its algal diet. Continuing with the eight-membered cyclic ethers, Kinnel and co-authors [56] described the presence of *cis*-dihydrorhodophytin (**27**) and *cis*-isodihydrorhodophytin (**28**) in the extract of *Aplysia brasiliana* Rang (accepted as *A. fasciata*), compound **27** behaving as an antifeedant in bioassays with swordtail fish.

**Figure 5:**
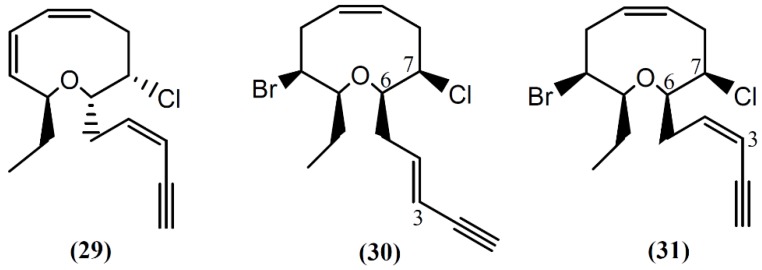
Nine-membered cyclic ether acetogenins isolated from sea hares of *Aplysia* genus.

Following spectral and X-ray diffraction studies, the same group identified brasilenyne (**29**) in the same sample (Figure 5), a nine-membered ether ring with fish-feeding deterrent activity [56]. The (+)-3*E*,6*R*,7*R*-Obtusenyne (**30**) and (+)-3*Z*,6*R*,7*R*-obtusenyne (**31**), two other halogenated acetogenins containing a nine-membered ether ring, were isolated from *A. dactylomela* [50]. As far as we know, there is no study concerning the bioactivity of these two compounds. More studies regarding the mechanism of action of this class of compounds are necessary for a better understanding of the bioactivities described.

## 4. Alkaloids

**Figure 6:**
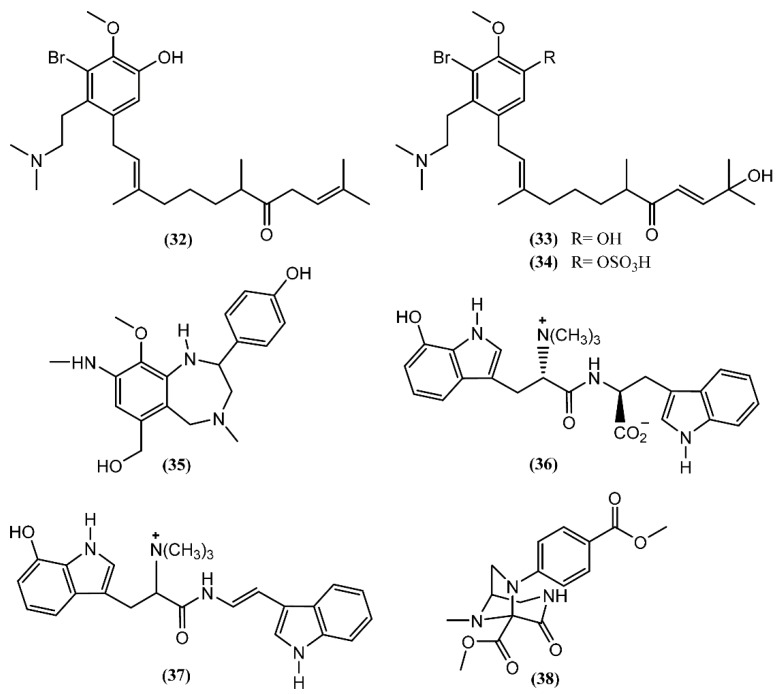
Alkaloids isolated from sea hares of *Aplysia* genus.

*Aplysia* chemical diversity and richness is also highlighted by the presence of alkaloids. Three prenylated aromatic compounds, aplaminone (**32**), neoaplaminone (**33**) and neoaplaminone sulfate (**34**)**,** were isolated from *A. kurodai* (Figure 6) [57]. The biogenesis of the aplaminones may be envisaged by coupling a sesquiterpenoid with a brominated dopamine moiety [57]. These compounds are cytotoxic to HeLa S_3_ cells *in vitro,* displaying IC_50_ values of 0.28, 1.6 × 10^−7^ and 0.51 mg/mL, respectively [57,58]. Based on the enantioselective synthesis of debromoneoaplaminone, Kigoshi *et al*. [59] established the absolute stereochemistry of aplaminone (**32**) and neoaplaminone (**33**). Another novel alkaloid, aplysepine (**35**), was isolated from *A. kurodai* by Ojika *et al.* [60]. The gross structure was elucidated, aplysepine (**35**) being the first 1,4-benzodiazepine alkaloid of marine origin. Moreover, the pattern of substitution of aplysepine (**35**) suggested a biosynthetic origin from anthranilic acid and β-phenylalanine, while other natural benzodiazepines are derived from anthranilic acid and α-amino acids. The analysis performed with a single specimen of *A. dactylomela* collected near Leigh Harbour (New Zealand) led to the isolation of dactylamides A and B (**36**–**37**) [61], two tryptophan-derived dipeptides with no significant activity reported until now. The absolute stereochemistry of compound **36** was determined by synthesis of deoxo-diastereomers and comparison of CD spectra [61]. Aplaminal (**38**) was another alkaloid isolated from a sea hare of the *Aplysia* genus [62]. This novel triazabicyclo[3,2,1]octane framework metabolite was found in the methanolic extract of *A. kurodai* and its stereostructure was confirmed by X-ray crystallographic analysis [62]. The 3,7,8-triazabicyclooctane skeleton has not yet been reported in a natural product, although the 1,2,3-triaminopropane framework of aplaminal (**38**) can be found in tetrahydrofolic acid [62]. According to this, Kuroda *et al*. [62] suggested a plausible biogenetic pathway for the carbon framework of aplaminal (**38**), based on the hydrolysis of the guanidine moiety of tetrahydrofolic acid, followed by oxidative cyclization. Compound **38** exhibited cytotoxicity against HeLa S_3_ cells, displaying an IC_50_ of 0.51 µg/mL [62]. Shortly afterwards, the first total synthesis of (−)-aplaminal was achieved in nine steps, with 19% overall yield [63].

## 5. Sterols and Degraded Sterols

**Figure 7:**
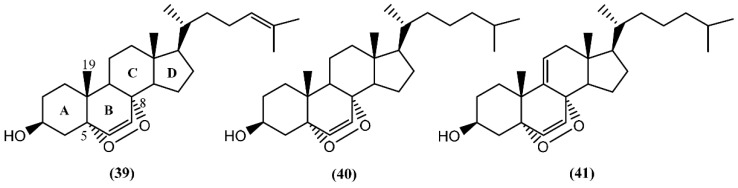
The 5α,8α-epidioxysterols from sea hares of *Aplysia* genus.

In the last decades, sterols from marine organisms such as fungus, macroalgae, sponges, corals and mollusks have shown a panoply of remarkable bioactivities, including antimicrobial [64], cytotoxic [65], antiviral [66], and antibacterial [67]. In the particular case of sterols from sea hares of the *Aplysia* genus, Jiménez and co-authors [68] described the presence of 5α,8α-epidioxy sterols (**39**–**41**) (Figure 7) in two species: *A. depilans* and *A. punctata*. Interestingly, some oxygenated sterols have also been reported from the egg masses of *Aplysia juliana* Quoy & Gaimard [69]. The endoperoxides (**39**–**41**) were isolated from the hepatopancreas (digestive gland) of *A. punctata*, whereas only compounds **40** and **41** were found in *A. depilans* [68]. The structure of compound **39** was confirmed by the synthesis from 25-hydroxycholesterol, in a multiple-step reaction [68]. Recently, Mun and colleagues [70] evaluated the cytotoxicity of 5α,8α-epidioxysterol (**39**) against human colorectal cancer cells (HCT 116), having obtained an IC_50_ value of 2.5 µM. Regarding the bioactivity of 5α,8α-epidioxycholest-6-en-3β-ol (**40**), Clark *et al*. [71] discovered antileishmanial properties, displaying an IC_50_ of 4.9 µM towards the amastigote form of *Leishmania donovani*, but no measurable activity against *Plasmodium falciparum*, *Trypanosoma cruzi*, and breast cancer cells (MCF-7).

**Figure 8:**
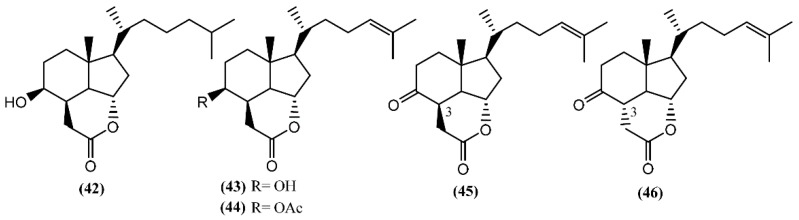
Degraded sterols from *Aplysia* sea hares containing the *cis*-hydrindane skeleton.

In addition to these epidioxy sterols, *Aplysia* mollusks are also known for the presence of families of compounds resulting from the degradation of marine steroids, entitled aplykurodins and aplykurodinones, which showed strong ichthyotoxic and cytotoxic properties [72,73]. The first degraded sterols to be discovered were aplykurodins A and B (**42**–**43**) (Figure 8), in *A. kurodai* [72]. Their carbon skeleton apparently derives from a dramatic oxidative degradation of the tetracyclic steroid nucleus, with the loss of ring-A carbon atoms and of the 19-methyl group (Figure 7) [74]. A very interesting structural feature in aplykurodins is the presence of a *cis*-hydrindane skeleton. Shoji and colleagues [75] showed that the sterols possessing a *cis*-C/D ring junction act as powerful inhibitors of histamine release from rat mast cells. In 1992, a study performed with the external parts of the body of *A. fasciata* led to the isolation of two ichthyotoxic lactones: 4-acetylaplykurodin B (**44**) and aplykurodinone B (**45**) [73]. Years later, Ortega and co-workers [76] isolated 3-*epi*-aplykurodinone B (**46**) from *A. fasciata* collected in Río San Pedro, Cadiz (Spain). Compound **46** was the last addition to the series, differing from aplykurodinone B (**45**) by the epimeric relationship at C-3 [76]. It was tested against mouse lymphoma (P-388), human lung carcinoma (A-549), human colon carcinoma (HT-29), and human melanoma (MEL-28) tumor cell lines, exhibiting mild *in vitro* cytotoxicity (ED_50_ of 2.5 µg/mL in all cases) [76].

**Figure 9:**
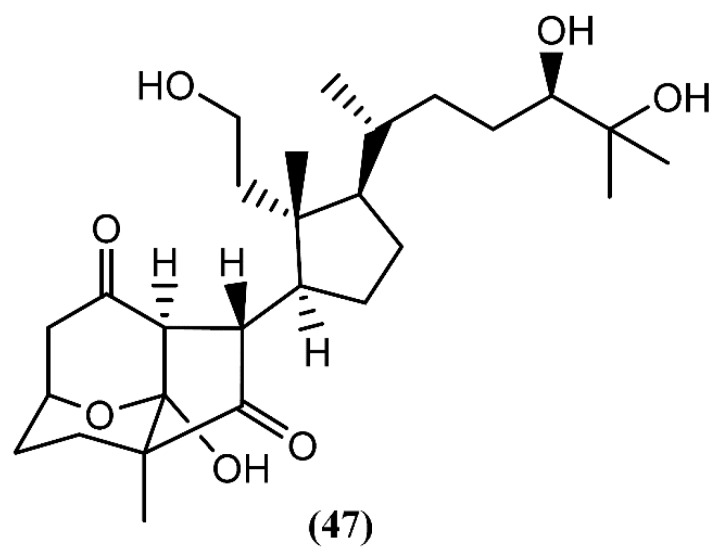
A 9,11-secosteroid from *A. kurodai* containing a tricyclic γ-diketone structure.

Recently, aplysiasecosterol A (**47**) (Figure 9), a new 9,11-secosteroid containing an unprecedented tricyclic γ-diketone structure, was isolated from *A. kurodai* [77]. A possible biosynthetic pathway for the tricyclic γ-diketone structure, having cholest-7-en-3*S*,5*R*,6*R*-triol as precursor, was suggested, attending to the structural similarity between both the cyclopentane ring and the side-chain part of aplysiasecosterol A (**47**) and those of known 9,11-secosteroids [77]. Compound **47** did not show significant cytotoxicity against HeLa S_3_ cells at 200 µM, but it exhibited moderate cytotoxicity against the human myelomonocytic leukemia (HL-60) cell line, displaying an IC_50_ of 16 µM [77]. Furthermore, this was the first molecule having this type of tricyclic ring system, which can be consider as a possible prototype for the synthesis of new analogues with interesting bioactivities.

## 6. Terpenoids

Terpenoids are widespread in algae, marine invertebrates, microorganisms, higher plants, arthropods and fungi [78]. Sea hares have traditionally been a source of terpenoids of dietary origin [79]. The close relationship between the compounds stored in the digestive glands of certain opisthobranchs of the genus *Aplysia* and the chemical constituents of the algae that form a major portion of their diet has been well established [36]. The genus *Aplysia* has been extensively studied, affording monoterpenes, sesquiterpenes, diterpenes and triterpenes with varied degrees of halogenation, accumulated from the algal diet and also modified by metabolic transformations [79,80]: the compounds from algae are occasionally chemically transformed by the sea hares [80], frequently being converted into less toxic compounds [81]. This is the main reason why some researchers focus on the study of the digestive gland of sea hares, aiming to discover new molecules to be used in the treatment of several diseases.

### 6.1. Monoterpenes

**Figure 10:**
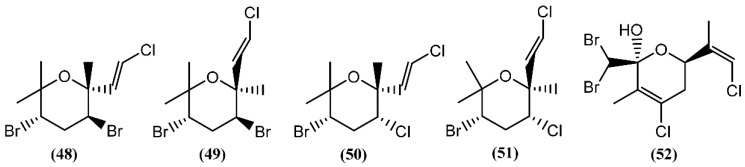
Pyrano-monoterpenes isolated from sea hares of the *Aplysia* genus.

Halogenated monoterpenes may be linear or cyclic. Generally, *Aplysia* species are known to yield numerous polyhalogenated monoterpenes, their origin essentially being in red algae of genera *Laurencia* and *Plocamium* on which the sea hares feed [82]. Cytotoxicity has been reported for a few marine monoterpenes in the past years. Four cytotoxins, aplysiapyranoids A–D (**48**–**51**) (Figure 10), having a halogenated tetrahydropyran, were isolated from the midgut gland of *A. kurodai* [83]. Compounds **48**–**51** exhibited moderate cytotoxicity against epithelial-like monkey kidney Vero cells, and Madin-Darby canine kidney (MDCK) and mouse melanoma (B16) cell lines, with IC_50_ values of 19–96 μg/mL [83]. Aplysiapyranoid D (**51**) displayed interesting activity against a human colorectal carcinoma cell line (Moser) (IC_50_ = 14 μg/mL) [83]. The total synthesis of aplysiapyranoid A (**48**), C (**50**) and D (**51**) was achieved from simple achiral allylic alcohols by Jung and co-workers [84,85,86], using a brominative cyclization reaction. Costatone (**52**), another polyhalogenated pyranoid monoterpene, was the main metabolite found in *A. parvula* [87]. It was previously described in *Plocamium costatum* (C. Agardh) Hooker & Harvey, a red algae which served as food for this sea hare [88,89,90,91].

**Figure 11:**
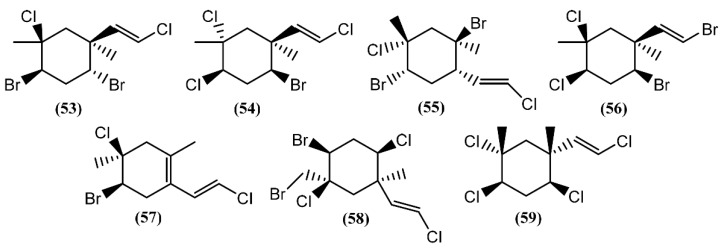
Cyclic monoterpenes isolated from sea hares of the *Aplysia* genus.

**Figure 12:**
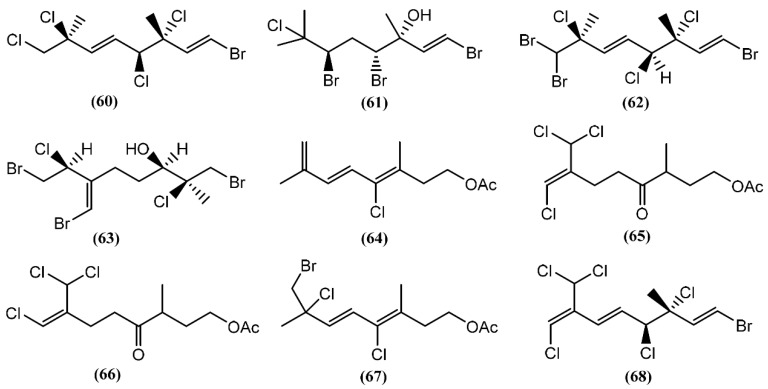
Linear monoterpenes isolated from sea hares of the *Aplysia* genus.

The halogenated monoterpenes (**53**–**57**) (Figure 11) isolated from Spanish specimens of *A. punctata* were all traced to the red alga *Plocamium coccineum* [92]. The 1*R*,2*S*,4*S*,5*R*-5-Chloro-2(*E*)-(chlorovinyl)-1,4-dibromo-1,5-dimethylcyclohexane (**55**) was also found in three specimens of *A. dactylomela* from San Juan de la Rambla, Tenerife (Spain), together with 4-bromo-5-bromomethyl-2,5-dichloro-1-(*E*)chloroethenyl-1-methylcyclohexane (**58**) [82]. Compounds **53** and **58** exhibited activity in a brine shrimp bioassay at 0.5 mg/mL, in the range of 90% and 100% lethality after 48 h, respectively [82]. Moreover, compound **58** was weakly cytotoxic towards human lung cancer (Lu1), human oral epidermoid carcinoma (KB), and hormone dependent human breast cancer (ZR-75-1) cells, displaying IC_50_ values of 12.9, 13.3, and 7.8 μg/mL, respectively. It also showed strong algicidal activity against the microalga *Chlorella fusca* and moderate antimicrobial activities [82]. The cyclic monoterpene aplysiaterpenoid A (**59**) (Figure 11) and the acyclic monoterpene aplysiaterpenoid B (**60**) (Figure 12) isolated from *A. kurodai* exhibited cytotoxic and ichthyotoxic properties [93]. Compound **59** showed mild cytotoxicity against various tumor cell lines, displaying IC_50_ values of 10 μg/mL for mouse lymphoma (L1210), 15.3 μg/mL for human lung carcinoma (QG-90) and 30.2 μg/mL for human breast cancer (MCF-7) [93]. Moreover, it showed significant ichthyotoxicity against mosquito fish *Oryzias latipes* and insecticidal activity against the German cockroach *Blatella germanica* and mosquito larvae *Anopheles gambiae* [94]. In addition to aplysiaterpenoid B (**60**), other linear monoterpenes were found in sea hares of the *Aplysia* genus (Figure 12). The compound 7-Chloro-3,7-dimethyl-1,4,6-tribromo-1-octen-3-ol (**61**) from *Aplysia californica* J.G. Cooper was the first of the series [95].

Besides compound **61**, one year later, the same group found 3,7-dimethyl-1,8,8-tribromo-3,4,7-trichloro-1,5-octadiene (**62**) [36,81] in the same species. However, as far as we know, there are no studies concerning to the bioactivity of compounds **61** and **62**. Kurodainol (**63**) was found in the gut of *A. kurodai*, its structure being determined by X-ray analysis [96]. *A. punctata* from Sancti Petri, Cádiz (Spain), contained four new unusual acetates of linear polyhalogenated monoterpenes (**64**–**67**) [97]. It was suggested that these acetates might be biotransformation products [97]. Compounds **65**–**67** showed identical cytotoxic properties against mice lymphoma (P-388) (ED_50_ = 2.5 μg/mL), human lung carcinoma (A-549) (ED_50_ = 1.5 μg/mL), human colon carcinoma (HT-29) (ED_50_ = 2.5 μg/mL) and human melanoma (MEL-28) (ED_50_ = 1.5 μg/mL) cell lines [97]. Another linear monoterpene, (1*E*,5*E*,7*E*)-1-bromo-7-dichloromethyl-3,4,8-trichloro-octa-1,5,7-triene (**68**), was isolated from *A. dactylomela* [82]. Compound **68** demonstrated significant cytotoxicity toward three cancer cell lines (HM02, HEP G2, and MCF 7), displaying IC_50_ values of 1.1, 1.0 and 1.5 μg/mL, respectively [82].

### 6.2. Sesquiterpenes

#### 6.2.1. Chamigrane Skeleton Sesquiterpenes

**Figure 13:**
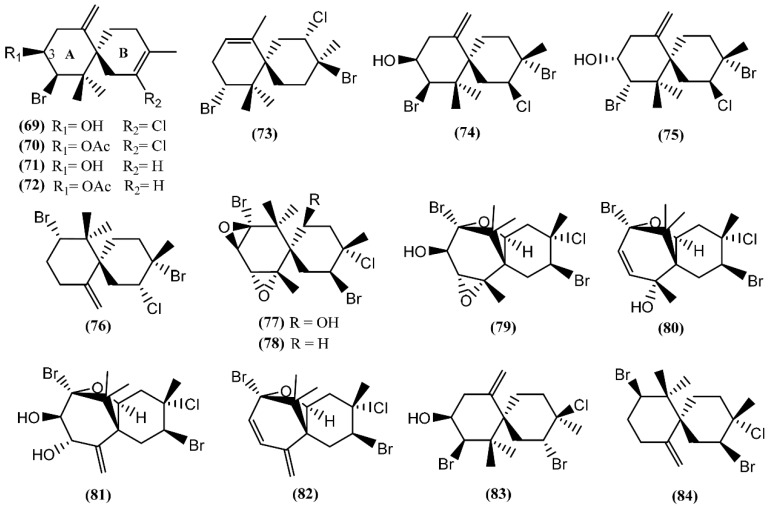
Chamigrane sesquiterpenes isolated from sea hares of *Aplysia* genus.

Halogenated chamigrane derivatives are regular sesquiterpenes with a spiro center [98]. Concerning the marine environment, secondary metabolites possessing this skeleton are characteristic of the red algae of the genus *Laurencia* and of the sea hare grazing on them [20]. Elatol (**69**), acetylelatol (**70**), deschlorelatol (**71**), acetyldeschlorelatol (**72**) and compound **73** (Figure 13) were isolated from the acetone extract of *A. dactylomela* digestive glands collected from the southwest coast of La Palma Island [99]. Elatol (**69**) has already displayed a diversity of biological activities, such as parasiticide against the amastigotes of *Trypanosoma cruzi* and *Leishmania amazonensis* [100,101], anti-tumor [102], antibacterial against some marine bacteria [103] and larvicidal against the dengue mosquito *Aedes aegypti* [104]. A recent study developed by Desoti *et al.* [105] found that the trypanocidal action of (−)-elatol might involve the induction of the autophagic and apoptotic death pathways triggered by an imbalance of the parasite’s redox metabolism. The cytotoxic effect of elatol (**69**) is due to its capacity to induce cell cycle arrest in the G_1_ and the sub-G_1_ phases, leading cells to apoptosis [102]. It is of note that elatol (**69**) and acetylelatol (**70**) showed cytotoxic activity against Vero cells, displaying IC_50_ values of 25 and 44.6 µM, respectively, while deschloroelatol (**71**) and its acetyl derivative (**72**) were inactive [99]. This result indicates the relevance of the chlorine atom and of the free hydroxyl group in C-3 of the A-ring to the cytotoxicity toward Vero cells. Moreover, elatol (**69**) and deschloroelatol (**71**) exhibited some antifungal properties against *Mycotypha microspore* and *Eurotium repens*, with compound **71** being more active [106]. In addition to compound **69**, Vairappan and co-authors [107] described the isolation of iso-obtusol (**74**) from *A. parvula*, while Díaz-Marrero *et al.* [108] reported the isolation of the chamigrenes iso-obtusol (**74**) and obtusol (**75**) from *A. dactylomela*. Compound **74** exhibited antibacterial activity against *Klebsiella pneumonia* and *Salmonella* sp., equalizing the potency of commercial antibiotics against these two bacteria [109]. Moreover, as compound **69**, iso-obtusol (**74**) also displayed *in vitro* and *in vivo* leishmanicidal activity [110]. On the other hand, compound **75** demonstrated significant cytotoxicity toward three cancer cell lines (HM02, HEP G2, and MCF 7) and moderate antimicrobial activity [82]. Recently, another chamigrene, named epi-obtusane (**76**)**,** has been isolated from an acetone extract of the digestive gland of *Aplysia oculifera* Adams & Reeve, showing *in vitro* cytotoxicity against different human cancer cell lines (PC-3, A549, MCF-7, HepG2 and HCT 116), with IC_50_ values in the low µg/mL range [111]. Continuing with chamigrane sesquiterpenes, Kaiser *et al.* [112] found prepacifenol and dehydroxyprepacifenol epoxides (**77**–**78**) in *A. dactylomela* collected from Brazilian waters. Prepacifenol epoxide (**77**) was previously reported in the digestive gland of *A. californica* [113]. This halogenated sesquiterpene diepoxide seems to be the precursor of johnstonol (**79**) [114], a chamigrane also found in *A. dactylomela* from Brazilian waters [115] and in the digestive gland of *A. californica* [36].

Pacifenol (**80**), the first natural product described to contain bromine and chlorine, was firstly found in the red algae *Laurencia pacifica* [116]. Pacifenol (**80**)**,** together with pacifenediol (**81**) and pacifidiene (**82**)**,** was isolated from *A. dactylomela* in 2001 [117]. Studies exploring the bioactivity of these chamigrenes showed that compound **80** presented a partial activity against the Gram-negative bacteria *Pseudomonas aeruginosa* [118] and 90% of mortality was registered after 24 h exposure to 23 µg/mL of pacifenol (**80**) in sea water during the brine shrimp bioassay [119]. Moreover, it is able to decrease the release of eicosanoids, with stronger potency on the cyclooxygenase (COX) pathway [120]. In 2014, Palaniveloo and Vairappan [20] studied the chemical relationship between red algae from the genus *Laurencia* and *A. dactylomela* from different geographical areas, having found a big diversity of chamigrenes, including rogiolol (**83**) which had been previously isolated from a *Laurencia* species [121]. Another study, involving two color variants of *A. dactylomela*, led to the isolation of nidificene (**84**) [122]. This compound was found to possess antiviral activity against herpes simplex virus-1 (HSV-1), the exo-methylene group being a key factor for the antiviral activity [123].

#### 6.2.2. Bisabolane and Cuparane Skeleton Sesquiterpenes

**Figure 14:**
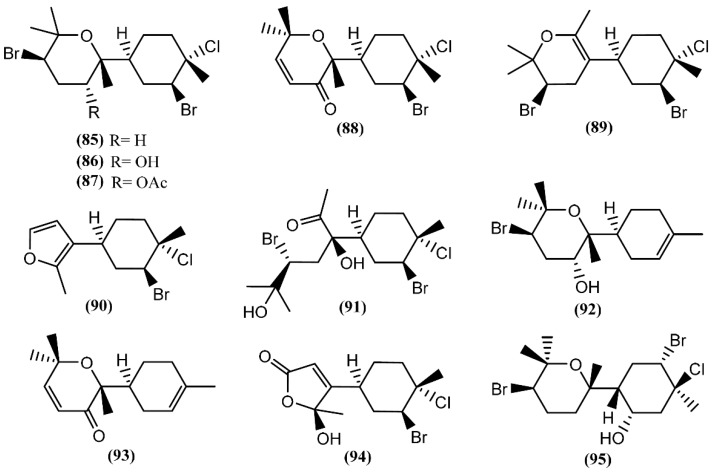
Bisabolane sesquiterpenes isolated from sea hares of the *Aplysia* genus.

The bisabolane skeleton develops from the cyclization of the geranyl cation and results in the formation of a monocyclic ring structure [124]. This type of compound appears to be among the simplest of sesquiterpenes, because of their structures; however, the bisabolane derivatives isolated from sea hares of the *Aplysia* genus contain a bicyclic ring structure (Figure 14). Six modified bisabolane sesquiterpenes, caespitane (**85**), caespitol (**86**), 8-acetylcaespitol (**87**), caespitenone (**88**), laucapyranoid A (**89**) and furocaespitane (**90**), were isolated from three distinct samples of *A. dactylomela* [82]. Compounds **85** and **86** exhibited activity in a brine shrimp bioassay in the range of 100% lethality within 24 h, dropping to 40% after 48 h [82]. Moreover, caespitol (**86**) displayed algicidal and nematocidal effects toward *Caenorrhabditis elegans* [82], and very weak cytotoxicity against HeLa 229 cells, presenting an IC_50_ of 100 µg/mL [125]. It is worth mentioning that small changes in chemical structures can have a pronounced influence on bioactivity. Accordingly, Wessels *et al.* [82] showed that 8-acetylcaespitol (**87**), which only differs from caespitol (**86**) in the acetyl group, was completely inactive in the same assays. Caespitenone (**88**) is an oxidation product of the algal metabolite caespitol (**86**) and is probably produced by the animal through the oxidation of the diet-derived natural product [126]. It was found to be active against HT29, MCF7 and A431 cell lines, exhibiting an IC_50_ of 18.9, 19.7 and 21.6 µM, respectively [127]. Studies regarding the bioactivity of laucapyranoid A (**89**) and furocaespitane (**90**) are necessary to evaluate the influence of the double-bound position in the tetrahydropyran ring and the impact of the furan ring, respectively. Another study with *A. dactylomela* led to the isolation of aplysiadactydiol (**91**), deschlorobromo caespitol (**92**), deschlorobromo caspitenone (**93**) and furocaespitanelactol (**94**) [128]. The same authors suggested compound **91** as a key intermediate for a unified biogenesis of regular and irregular marine algal bisabolene-type metabolites that are also present in sea hares [128]. Deodactol (**95**), an antineoplastic sesquiterpene isolated from *A. dactylomela*, was moderately cytotoxic to the L1210 cell line, with an ED_50_ of 12 µg/mL [129].

**Figure 15:**
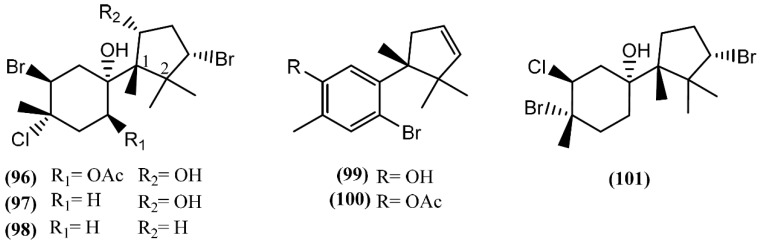
Cuparane sesquiterpenes isolated from sea hares of the *Aplysia* genus.

Cuparane sesquiterpenes are present in some marine organisms, liverworts and higher plants [130,131]. In those metabolites comprising a cuparane core, carbons 1 and 2 from the aliphatic portion are substituted with a methyl group and dimethyl groups, respectively (Figure 15) [98]. *A. dactylomela*, collected at the Eastern Cape coast of South Africa, yielded algoane (**96**), 1-deacetoxyalgoane (**97**) and 1-deacetoxy-8-deoxyalgoane (**98**) (Figure 15) [122]. Two other cuparane sesquiterpenes with unprecedented oxygenation patterns, namely cupalaurenol (**99**) and cupalaurenol acetate (**100**), were isolated from *A. dactylomela* [132]. Since cupalaurenol (**99**) is reported in algae of the *Laurencia* genus, the acetylation of compound **99** seems to occur inside the sea hare [133]. Compound **99** exhibited potent inhibition against *Staphylococcus* sp., *Staphylococcus* *aureus*, and *Salmonella* sp., presenting a minimum inhibitory concentration (MIC) of 125 µg/mL for all strains [133]. More recently, oculiferane (**101**) was isolated from the digestive gland of *A. oculifera* [111]. Compound **101** showed the same *in vitro* cytotoxicity reported for *epi*-obtusane (**76**), being significantly more active against A549 cells [111].

#### 6.2.3. Laurane Skeleton Sesquiterpenes

**Figure 16:**
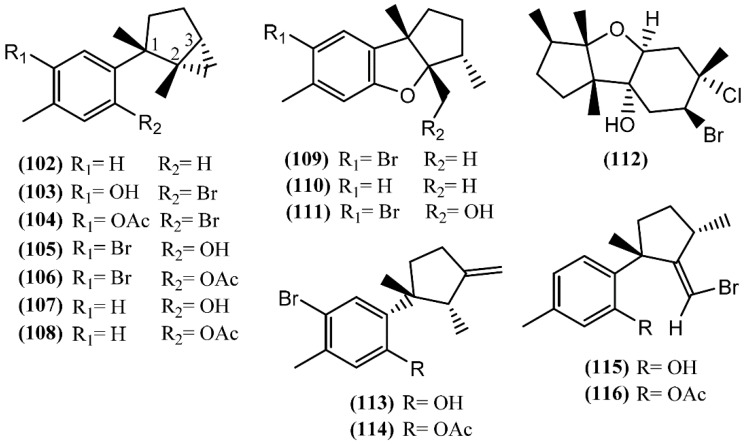
Laurane sesquiterpenes isolated from sea hares of the *Aplysia* genus.

In Laurane-type compounds, the three methyl groups in the aliphatic portion are located at positions 1, 2 and 3 [98]. The *Laurencia* genus is considered to be the main producer of laurane-type sesquiterpenes among marine organisms in general. Since *Aplysia* sea hares feed on this algae, as reported above, it is not strange that this class of compounds is one of the most representative in *Aplysia* spp. A single specimen of *A. dactylomela* collected at Kohama Island, Okinawa, provided cyclolaurene (**102**), cyclolaurenol (**103**) and cyclolaurenol acetate (**104**) (Figure 16) [132]. The structures, including their absolute configurations, were elucidated from spectroscopic data and chemical interconversion. Compounds **102**–**104** displayed antibacterial, antifungal and ichthyotoxic activities [132]. Bioassay-guided purification of the methanolic extract of *A. kurodai* afforded four active compounds, which were identified as laurinterol (**105**), laurinterol acetate (**106**), debromolaurinterol (**107**), and debromolaurinterol acetate (**108**) by spectroscopic analysis [134]. Compound **105** had been previously identified by Stallard and Faulkner [36,81] and by Findlay and Li [44] in *A. californica* and *A. punctata*, respectively, and it was considered as a promising substance for the prevention and inhibition of melanoma, since it can inhibit the growth of melanoma cells by inducing apoptosis, displaying an IC_50_ of 10 µg/mL [135]. Regarding antibacterial activity, compounds **105** and **107** are more potent than the respective acetates (**106** and **108**) [134]. Moreover, debromolaurinterol (**107**), laurinterol (**105**) and its acetate (**106**) showed moderate cytotoxicity against HeLa cells, displaying IC_50_ values of 18, 32 and 20 µg/mL, respectively [134]. In 1963, Yamamura and Hirata [21] elucidated the structures of aplysin (**109**), debromoaplysin (**110**) and aplysinol (**111**) isolated from *A. kurodai*. Aiming to study the effects of compound **109** on the inhibition of gastric cancer, Liu and colleagues [136] showed that it can inhibit the proliferation and induce apoptosis of SGC-7901 cells *in vitro*. In addition, Gong *et al*. [137] demonstrated that aplysin (**109**) can enhance the effect of temozolomide (a chemotherapy drug) on glioma cells by increasing miR-181 expression. According to this, a recent study showed that aplysin (**109**) can actually induce apoptosis in glioma cells by interference with the HSP90/AKT pathway [138]. Moreover, a toxicological study suggested that aplysin (**109**) has a significant protective effect on hepatic injury in ethanol-treated rats by modulating the ethanol-metabolizing pathway, attenuating oxidative stress, ameliorating mitochondrial function, and inhibiting mitochondrial damage-mediated apoptosis, which ultimately prevent and repair alcoholic liver injury [139]. Ibhayinol (**112**) was found in a South African *A. dactylomela* [122]. Copley *et al*. [140] established its absolute stereochemistry based on a single-crystal X-ray diffraction experiment.

Allolaurinterol (**113**), isolaurenisol (**115**) and their respective acetates (**114** and **116**) were isolated from a crude organic extract of *A. dactylomela*, collected near Leigh Harbour, Northland (New Zealand) [61]. Compounds **113** and **115** had previously been reported in *Laurencia obtusa* and in *Laurencia distichophylla*, respectively [141,142], but their corresponding acetates **114** and **116** were firstly prepared by synthesis [143]. Appleton and co-authors [61] isolated the acetates from a natural source, indicating that this acetylation probably occurs in the digestive gland of the sea hare. Studies involving the biological activity of these compounds showed that the sesquiterpenes **113** and **115**, containing the free phenol group, exhibited moderate P388 and BSC-1 cytotoxicity, also revealing significant antibacterial activity against *Bacillus subtilis* and moderate activity against the fungus *Trichophyton mentagrophytes*. On the other hand, the corresponding acetate derivatives (**114** and **116**) displayed lower P388 cytotoxicity and were less effective against *B. subtilis* [61].

#### 6.2.4. Brasilane and Omphalane Skeleton Sesquiterpenes

**Figure 17:**
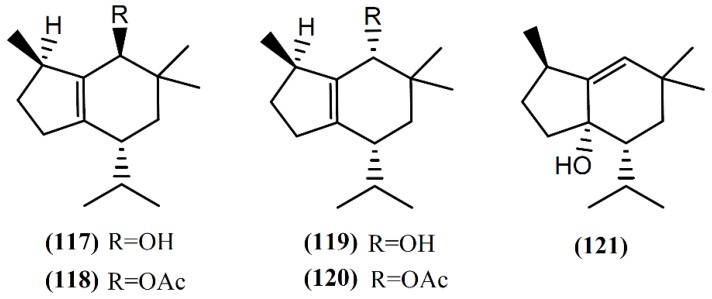
Brasilane sesquiterpenes isolated from sea hares of the *Aplysia* genus.

The brasilane skeleton has the basic structure of octahydro-1,6,6-trimethyl-4-(1-methyl)-1*H*-indene [98]. Two non-halogenated sesquiterpenes (Figure 17), brasilenol (**117**) and brasilenol acetate (**118**), have been isolated along with epibrasilenol (**119**), from the digestive glands of *A. brasiliana* collected along the coast of Texas [144]. In addition to these brasilane-type sesquiterpenes, Ioannou and colleagues [43] isolated epibrasilenol acetate (**120**) and 6-hydroxy-1-brasilene (**121**) from the organic extract of digestive and hermaphroditic glands of *A. punctata*. The structure of brasilenols was established by detailed spectral analysis and limited chemical conversions. The methyl, isopropyl, and hydroxyl groups and the tetrasubstituted double bond were defined by spectral analysis [43]. As far as we are aware, the bioactivity of these compounds has not been reported yet.

**Figure 18:**
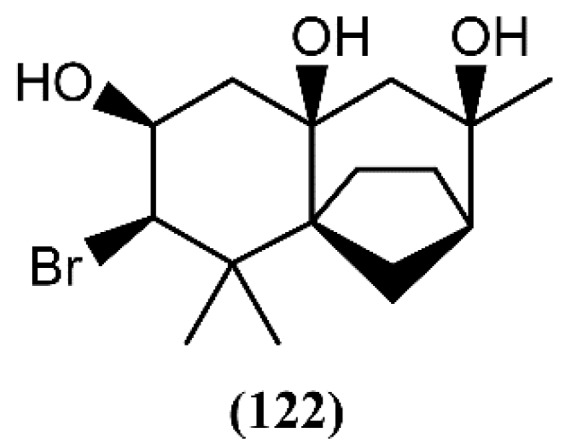
First marine omphalane-derived sesquiterpene, isolated from *A. dactylomela*.

A few years ago, dactylomelatriol (**122**), the first naturally occurring omphalane-derived sesquiterpene from the marine environment, was extracted from *A. dactylomela* [108] (Figure 18). This skeleton was encountered for the first time in a liverwort [145] and its structure and relative configuration were established by spectroscopic evidence [108]. According to its structure, Diaz-Marrero and colleagues [108] proposed a biogenetic route, from deschloroelatol (**71**) until the formation of the rhodolaurane skeleton intermediate, which can undergo a 1,2-shift to generate the omphalane ring system. Compound **122** was tested for its antimicrobial capacity, but revealed to be inactive [108]. However, more studies involving different assays and bioactivities are necessary and could lead to promising results.

#### 6.2.5. Eudesmane and Snyderane Skeleton Sesquiterpenes

**Figure 19:**
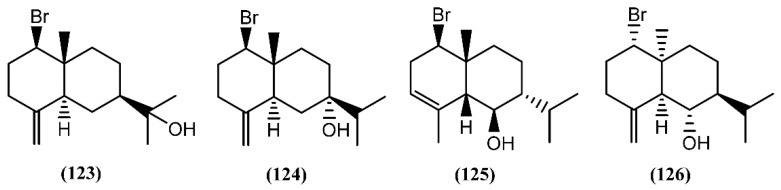
Eudesmane sesquiterpenes isolated from sea hares of the *Aplysia* genus.

Eudesmane-type sesquiterpenes (Figure 19), formerly referred to as selinanes, have been recognized in several terrestrial and marine organisms, and occasionally encountered in *Aplysia* [146]. Two halogenated eudesmanes, brasudol (**123**) and isobrasudol (**124**), were found in the digestive gland of *A. brasiliana* from Florida [146]. These brominated eudesmanes have the same absolute configuration of the natural β-eudesmol and showed feeding-deterrent activity [146]. While compounds **123** and **124** were not reported in the *Laurencia* genus, other species, such as *Laurencia filiformis* (C.Agardh) Montagne [147], *Laurencia nidifica* J.Agardh [148] and *Laurencia nipponica* Yamada [149], were found to produce similar sesquiterpenes, apparently modified by the sea hares. Later, two antipodal *cis*-fused eudesmanes, lankalapuol A (**125**) and B (**126**), were found in two independent collections of *A. dactylomela* collected in Hawaii and Sri Lanka [150]. Since the concomitant production of antipodal compounds from a single organism is unusual, it was hypothesized that both sesquiterpenes derived from sea hares’ algal diet.

**Figure 20:**
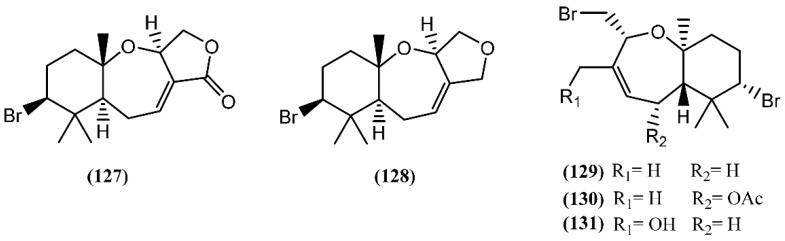
Snyderane sesquiterpenes isolated from sea hares of the *Aplysia* genus.

Snyderane sesquiterpenes are bromo monocyclo-nerolidol derivatives [98]. A collection of *A. dactylomela* from the Sepanggar Island demonstrated to be a great source of snyderane sesquiterpenes, affording aplysistatin (**127**), palisadins A (**128**) and B (**129**), 5-acetoxypalisadin B (**130**) and 12-hydroxypalisadin (**131**) (Figure 20) [151]. The analysis of its diet, namely *Laurencia snackeyi* (Weber-van Bosse) M.Masuda, showed the presence of these compounds, except 12-hydroxipalisadin (**131**) [107], suggesting that its biogenesis by hydroxylation of palisadin B (**129**) probably happens in the gut of the sea hare. The antileukemic agent aplysistatin (**127**) was firstly isolated from the 2-propanol extract of *A. angasi* [152]. A study regarding the effects of the algal diet on the susceptibility of *A. parvula* to predation indicated that these palisadins appear to be ineffective in the defense against some fishes [153]. However, the three halogenated compounds **127**, **128** and **130** exerted profound inhibitory effects on nitric oxide (NO) production by lipopolysaccharide (LPS)-stimulated RAW 264.7 cells [154]. Further experiments demonstrated that, in addition to NO, compound **127** also inhibited prostaglandin-E2 (PGE2) production [154]. This activity was attributed to the modulation of anti-inflammatory agents via the inhibition of nitric oxide synthase (NOS) and cyclooxygenase-2 (COX-2) expression [154].

### 6.3. Diterpenes

**Figure 21:**
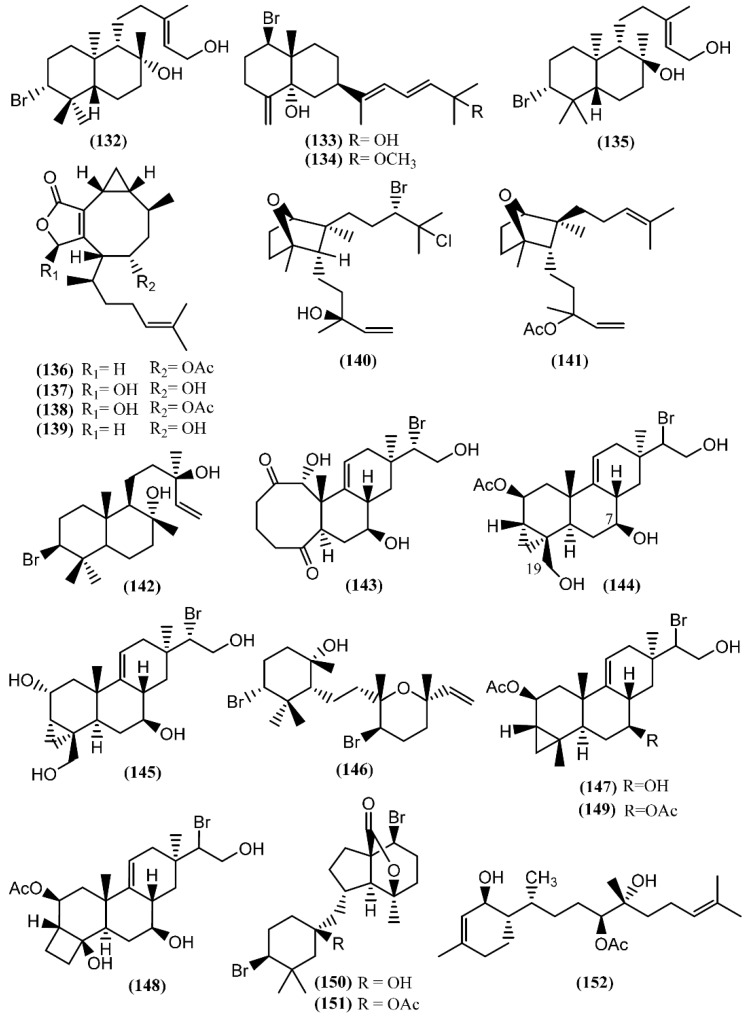
Diterpenes isolated from sea hares of the *Aplysia* genus.

Marine diterpenes have been isolated from both animals and plants. Although red algae of the genus *Laurencia* mainly produce bromine-containing metabolites with sesquiterpenic skeletons, there are several examples of bromine diterpenes as well. The first halogenated diterpenoid, aplysin-20 (**132**) (Figure 21), was found in *A. kurodai* [155]. Further studies on the same species led to the brominated diterpene aplysiadiol (**133**), together with the methyl ether derivative (**134**), which contains a rare extended sesquiterpenic skeleton of a prenylated eudesmane [156]. The presence of compound **133** in *Aplysia* has a probable origin in the diet, as it was also found in the red algae *Laurencia japonensis* T.Abe & Masuda [157]. Compound **133** exhibited a potent effect against *Staphylococcus* sp., *S. aureus* and *Salmonella* sp. [133]. Studies involving *A. kurodai* yielded *epi*-aplysin-20 (**135**), an epimer of **132** [158].

Non-halogenated diterpenoids, namely acetoxycrenulide (**136**), 1,9-dihydroxycrenulide (**137**), 1-hydroxy-9-acetoxycrenulide (**138**) and 9-hydroxycrenulide (**139**), were found in the digestive glands of Pacific *Aplysia vaccaria* Winkler [159]. This series of crenulides is typical of the brown alga *Dictyota crenulata* J.Agardh [160]. One of them, acetoxycrenulide (**136**), is recognized to be highly toxic to the herbivorous reef fish *Eupomacentrus leucostictus* at very low concentrations (10 µg/mL) [160], and to present anti-microfouling properties against two marine bacterial strains, 4M6 and D41, displaying an EC_50_ of approximately 69 and 82 µM, respectively [161]. Moreover, studies regarding the cytotoxicity of compound **136** showed moderate activity against human nasopharynx carcinoma (KB), human lung carcinoma (NSCLC-N6), murine leukemia (P-388), and murine leukemia expressing multi-drug-resistance gene (P-388/DOX) cells [162]. An anti-melanogenesis study demonstrated that 1,9-dihydroxycrenulide (**137**) inhibits melanin synthesis in B16F10 melanoma cells, pointing to a possible therapeutic use in the treatment of hyperpigmentation [163].

A new bicyclic diterpene, dactylomelol (**140**), was first isolated from *A. dactylomela* [164] and found afterwards in specimens of *Laurencia* sp. [165]. Compound **140** exhibited activity in a brine shrimp bioassay in the range of 100% lethality within 24 h to 40% after 48 h [82]. Since this discovery, another diterpene sharing the same bicyclic system as **140**, named punctatene acetate (**141**), was isolated from *A. punctata*, together with isoconcinndiol (**142**), neopargueroldione (**143**), parguerol (**144**), deacetylparguerol (**145**) and punctatol (**146**) [44]. Parguerol (**144**) and other two derivatives, deoxyparguerol (**147**) and isoparguerol (**148**), were previously found in *A. dactylomela* [166]. Compounds **144** and **148** induced neurite outgrowth in rat pheochromocytoma PC-12 cells at concentrations of 25 and 50 µM, respectively [167], pointing to a possible use in the treatment of various neuronal degenerative disorders. It of note that deoxyparguerol acetate (**149**), isolated from the methanolic extract of *A. kurodai* collected from Toyama Bay in the Japan Sea, did not show the same capacity of parguerol (**144**)**,** even at a concentration of 100 µg/mL [167], suggesting that the free hydroxyl group at C-7 and/or the presence of the hydroxyl group at C-19 is essential to the reported bioactivity. According to this, an *in vitro* screening on Ehrlich carcinoma tumor cells demonstrated that isoparguerol (**148**) is slightly more effective than other parguerol derivatives, while deoxyparguerol derivatives are the least active [168]. In addition, contrary to the effect of acetylation on the neurotrophic activity in PC-12 cells, the acetyl derivatives of these compounds have high cytotoxic activity, the number of acetoxy groups being essential for maximum cytotoxicity [168].

The dibromoditerpene designated angasiol (**150**) was isolated from a 2-propanol extract of *Aplysia angasi* G.B. Sowerby II (accepted as *A. dactylomela*) [169]. Years later, the corresponding acetate (**151**) was found in *A. juliana* from the Karachi coastline of the Arabian ocean [170].

Recently, dactyloditerpenol acetate (**152**), a new regular diterpene possessing an unusual 1,6-anti-3-methylcyclohex-2-en-1-ol ring system, was found in *A. dactylomela* [171]. Kishi’s method was applied to assign the absolute configuration of the hydroxyl groups [171]. *In vitro* studies involving this prenylbisabolane-type diterpene (**152**) revealed its potent capacity to inhibit thromboxane B_2_ and the superoxide anion generated in LPS-activated rat neonatal microglia, displaying IC_50_ values of 0.4 and 1 µM, respectively [171]. These results are indicators for a possible therapeutic approach to ameliorate neuroinflammatory disorders [172].

### 6.4. Triterpenes

**Figure 22:**
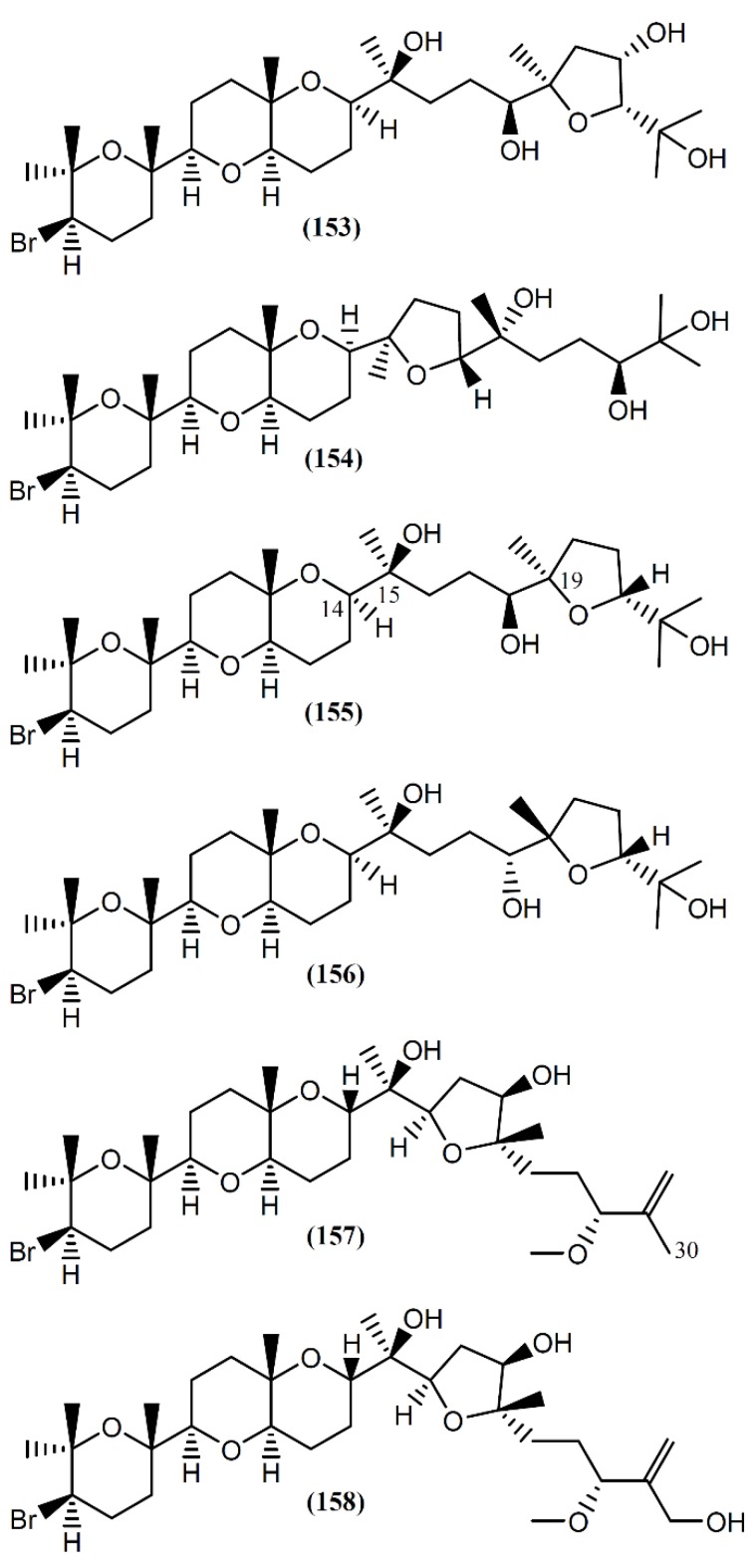
Brominated triterpenes with tetracyclic skeletons isolated from *A. dactylomela*.

The interest in the biological activity of marine triterpenoids continues, with recent works focusing on their antiviral [173], anti-inflammatory [174], and antitumor [175,176,177] capacity. Two studies described the isolation of triterpenoids from *Aplysia* species [178,179]. Aplysiol A (**153**) and B (**154**) (Figure 22) are interesting squalene-derived polyethers containing a dioxabicyclo[4,4,0]decane ring, which were isolated together with structurally related metabolites, namely thyrsiferol (**155**) and venustatriol (**156**), from a South China Sea collection of *A. dactylomela* [178]. Posteriorly, Ola *et al.* [180] and Cen-Pacheco *et al.* [181] revised the structure of Aplysiol B (**154**). Compound **155** and the antiviral **156** were previously reported as metabolites of *Laurencia thyrsifera* J.Agardh [182] and *Laurencia venusta* [183], respectively. Aplysiol A (**153**) and B (**154**) were present on the mantle of the sea hare, suggesting an involvement of these molecules in the chemical defense mechanism of the mollusk. Strongly supporting their possible defensive role, Manzo *et al.* [178] demonstrated that compounds **153** and **154** were active in the feeding-deterrence test against gold fish *Carassius auratus* at 50 µg/cm^2^, and were toxic in the ichthyotoxicity assay on *G. affinis* at a concentration of 10 ppm. Thyrsiferol (**155**) showed a potent and selective activity against P-388 cells, displaying an IC_50_ of 0.01 µg/mL [184]. It also presented moderate cytotoxicity against A549 and HT29 cells, with an IC_50_ of 10 µg/mL for both tumor cell lines [184]. Interestingly, degradation studies involving oxidative fragmentation of thyrsiferol (**155**) produced subunits devoid of significant cytotoxic activity [184]. Moreover, molecular modeling studies have proposed that the presence of the flexible chain around C-14 to C-19 is one of the essential factors related to the cytotoxic activity of this compound, whereas the presence or absence of the hydroxyl group at C-15 is indifferent [184,185]. More recently, *in vitro* studies using human breast tumor (T47D) cells showed that thyrsiferol (**155**) inhibits the activation of hypoxia-inducible factor-1 (HIF-1), a transcription factor with an important role in the etiology and progression of cancer, by interference from various mechanisms, such as the metabolism of anaerobic energy, angiogenesis and drug resistance [186,187]. In addition to the cytotoxic activity by apoptosis induction, thyrsiferyl derivatives are also recognized by distinct biological activities, including the selective inhibition of serine/threonine phosphatase 2A, a protein implicated in cell growth and signaling [188,189].

Aplysqualenols A (**157**) and B (**158**), brominated triterpenes structurally related to thyrsiferol, were isolated from two specimens of *A. dactylomela* collected in Puerto Rico [179]. A biogenetic pathway for compound **157** was suggested, compound **158** being posteriorly obtained by enzymatic hydroxylation at C-30 [179]. Upon screening by the National Cancer Institute’s (NCI’s) *in vitro* antitumor assay, containing 60 human tumor cell lines, the squalene-derived polyether **157** exhibited inhibitory activity against SNB-19 Central Nervous System (CNS) cancer cells and T-47D breast cancer cells, with IC_50_ values of 0.4 and 0.3 µg/mL, respectively [179]. Furthermore, aplysqualenol A (**157**) revealed antiviral properties against herpes simplex virus type 1 (HSV-1) and type 2 (HSV-2), varicella zoster virus (VZV) and human cytomegalovirus (HCMV), and remarkable toxicity against Epstein-Barr virus (EBV), with no accompanying toxicity seen in the host Daudi cells [179]. Regarding antiplasmodial activity, compounds **157** and **158** showed moderate effect toward *Plasmodium falciparum*, with IC_50_ values of 11 and 18 µg/mL, respectively [179].

## 7. Other Metabolites

**Figure 23:**
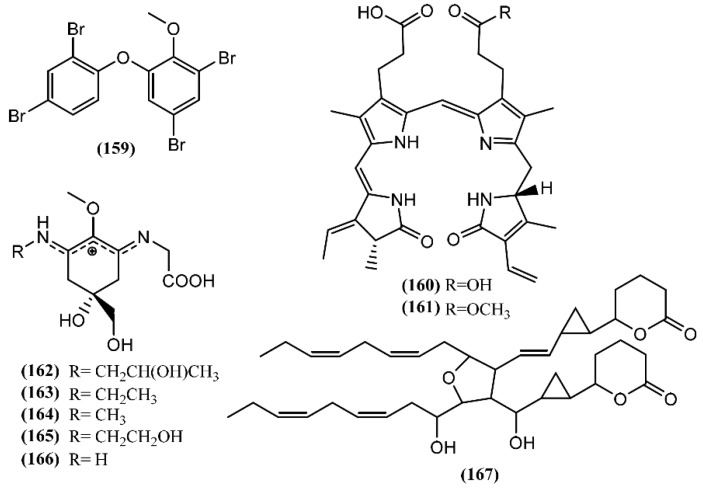
Other metabolites isolated from sea hares of the *Aplysia* genus.

A polybrominated diphenyl ether (**159**) (Figure 23) was detected in the green alga *Cladophora fascicularis* and isolated from the digestive gland of *A. dactylomela* [190]. Compound **159** exhibited potent anti-inflammatory properties and antibacterial activity against *Escherichia coli*, *Bacillus subtilis*, and *Staphylococcus aureus* [190].

Bioassay-guided fractionation led to the isolation of two purple molecules, namely phycoerythrobilin (**160**) and aplysioviolin (**161**), from the ink of *A. californica* [191]. Compound **161** was also found in *A. fasciata*, *A. dactylomela* and *A. parvula* [61,119]. Sea hares produce compound **160** and **161** from phycoerythrin, a photosynthetic pigment found in their red-alga diet: the cleavage in the digestive gland occurs to generate phycoerythrobilin (**160**), followed by a methylation in the ink gland to give aplysioviolin (**161**) [191,192]. Compounds **160** and **161** revealed to be deterrents against predatory blue crabs, being equally effective at 6.25 mg/mL [192]. This work developed by Kamio and co-authors [192] was the first demonstration of an animal converting a photosynthetic pigment into a chemical defense. It is of note that *in vitro* assays demonstrated that phycoerythrobilin (**160**) at 1 µmol/L significantly suppressed IgE antigen-stimulated degranulation of RBL-2H3 cells, while phycoerythrin had no effect [193]. Additionally, the suppression of mast cell degranulation was also observed in rats treated orally with compound **160** at 1 µmol/kg, suggesting interesting anti-inflammatory properties [193].

In 2005, HPLC analysis performed by Przeslawski *et al.* [194] allowed the detection of different mycosporine-like amino acids (MAAs) in egg masses of *Aplysia* spp. More recently, three new mycosporine-like amino acids (MAAs), namely aplysiapalythines **A**–**C** (**162**–**164**) were isolated, together with asterina 330 (**165**) and palythine (**166**), from opaline, a glandular component of the defensive ink secretion of *A. californica* collected from waters off southern California [195]. The primary mycosporine-glycine is synthesized from 3-dehydroquinate by the shikimate pathway and posteriorly transformed through chemical and/or biochemical conversions into other secondary MAAs [196]. It is important to note that macroalgae, fungi and cyanobacteria biosynthesize MAAs, while animals, such as *Aplysia* sea hares, which lack the shikimate pathway, acquire these metabolites from their diet or from symbionts [196,197,198]. Multiple properties are attributed to MAAs, such as the capacity to protect against UV radiation (e.g., MAAs are used in the chemical defense of *Aplysia* spp. egg masses as potential sunscreens) [194,199,200,201,202], and antioxidant [203,204] and osmotic regulation [205]. However, sea hares can modify the function of MAAs: these metabolites are provided from the algae diet, being concentrated in the defensive sea hare secretion, as well as in their skin, functioning as intraspecific alarm cues [195,197].

A particular polyketide with a planar structure, called aplydilactone (167), was isolated from the lipophilic extract of *A. kurodai* and considered as a new dimeric fatty acid metabolite [190]. According to its molecular structure, Ojika and colleagues [206] suggested that its biosynthesis involves two molecules of eicosapentaenoic acid that undergo unsymmetrical dimerization and oxidative cyclization to form lactones and cyclopropanes [207]. *In vitro* studies developed by the same group demonstrated that aplydilactone displayed capacity to activate phospholipase A2, an enzyme involved in the inflammatory process [206].

## 8. Conclusions

**Figure 24:**
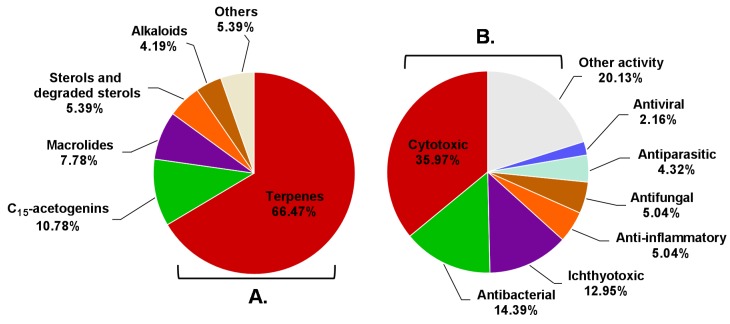
Distribution of compounds described in *Aplysia* spp. among the several chemical classes (**A**) and the prevalence of bioactivities attributed to them (**B**).

According to the above information, *Aplysia* sea hares have provided an important playing field for natural product chemists. To the best of our knowledge, only the species reported in this manuscript have been chemically characterized, these animals being a source of a wide diversity of secondary metabolites with interesting biological properties. Terpenes were the most represented structural class, with more than half of *Aplysia* secondary metabolites (111 MNPs) (Figure 24A), most of them probably obtained from the algal diet. However, some terpenes can also be a product of the modification of *Aplysia* dietary compounds. Other algae-related metabolites, namely C_15_-acetogenins, rate second in proportion (10.78%). Of the 167 reported molecules, 102 (61.08%) were found to be bioactive. However, this does not mean that the remaining 38.92% are inactive. Indeed, their bioactivity may be revealed in future studies. Attending to the number of activities attributed to each compound, they can be bi-active, tri-active and tetra-active. As such, and to get a general overlook of the activity spectra of *Aplysia* spp. molecules, one must count them two, three or four times, respectively. By doing so, seven main bioactivities can be highlighted (Figure 24B), cytotoxicity being the main one. 

Indeed, the discovery of particular macrolides, namely aplyronines, was shortly accompanied by strong evidence of their anti-cancer potential and mechanism of action. It is of note that not only macrolides were discovered, but also halogenated terpenoids with cytotoxic, antibacterial, antifungal, antiviral and anti-inflammatory properties.

**Figure 25:**
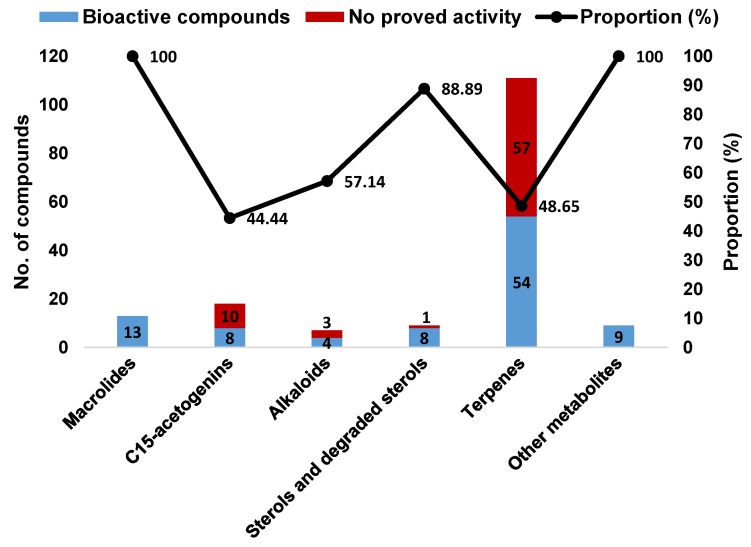
Bioactive compounds from sea hares of the *Aplysia* genus according to the chemical class.

A closer look at the bioactivity reports within each structural class so far reveals distinct potential (Figure 25). The 13 *Aplysia* spp. macrolides were all active. Although only 48.65% of the described terpenes displayed some activity, this seems to actually be the most interesting class because of the large number of metabolites, yielding more candidates for further exploitation.

In general, most of the secondary metabolites found in *Aplysia* sea hares have displayed interesting *in vitro* cytotoxic properties, which may provide useful leads for anti-cancer drugs. However, additional pharmacological testing is required to determine whether the cytotoxicity observed for these marine compounds resulted from a specific pharmacologic effect rather than a general toxic effect on the tested cancer cell lines. This should be a prerequisite to understand which molecules may constitute potential drug leads for the treatment of several diseases.
